# Geminin-Deficient Neural Stem Cells Exhibit Normal Cell Division and Normal Neurogenesis

**DOI:** 10.1371/journal.pone.0017736

**Published:** 2011-03-09

**Authors:** Kathryn M. Schultz, Ghazal Banisadr, Ruben O. Lastra, Tammy McGuire, John A. Kessler, Richard J. Miller, Thomas J. McGarry

**Affiliations:** 1 Feinberg Cardiovascular Research Institute, Northwestern University Feinberg School of Medicine, Chicago, Illinois, United States of America; 2 Department of Cell and Molecular Biology, Northwestern University Feinberg School of Medicine, Chicago, Illinois, United States of America; 3 Robert H. Lurie Cancer Center, Northwestern University Feinberg School of Medicine, Chicago, Illinois, United States of America; 4 Department of Molecular Pharmacology and Biological Chemistry, Northwestern University Feinberg School of Medicine, Chicago, Illinois, United States of America; 5 Department of Neurology, Northwestern University Feinberg School of Medicine, Chicago, Illinois, United States of America; University of South Florida, United States of America

## Abstract

Neural stem cells (NSCs) are the progenitors of neurons and glial cells during both embryonic development and adult life. The unstable regulatory protein Geminin (*Gmnn*) is thought to maintain neural stem cells in an undifferentiated state while they proliferate. Geminin inhibits neuronal differentiation in cultured cells by antagonizing interactions between the chromatin remodeling protein Brg1 and the neural-specific transcription factors Neurogenin and NeuroD. Geminin is widely expressed in the CNS during throughout embryonic development, and Geminin expression is down-regulated when neuronal precursor cells undergo terminal differentiation. Over-expression of Geminin in gastrula-stage Xenopus embryos can expand the size of the neural plate. The role of Geminin in regulating vertebrate neurogenesis in vivo has not been rigorously examined. To address this question, we created a strain of *Nestin-Cre/Gmnn^fl/fl^* mice in which the Geminin gene was specifically deleted from NSCs. Interestingly, we found no major defects in the development or function of the central nervous system. Neural-specific *Gmnn^Δ/Δ^* mice are viable and fertile and display no obvious neurological or neuroanatomical abnormalities. They have normal numbers of BrdU^+^ NSCs in the subgranular zone of the dentate gyrus, and *Gmnn^Δ/Δ^* NSCs give rise to normal numbers of mature neurons in pulse-chase experiments. *Gmnn^Δ/Δ^* neurosphere cells differentiate normally into both neurons and glial cells when grown in growth factor-deficient medium. Both the growth rate and the cell cycle distribution of cultured *Gmnn^Δ/Δ^* neurosphere cells are indistinguishable from controls. We conclude that Geminin is largely dispensable for most of embryonic and adult mammalian neurogenesis.

## Introduction

All neurons and glial cells in the brain are derived from neural stem cells (NSCs). NSCs maintain their own numbers by self-renewal and also give rise to daughter cells that terminally differentiate into neurons, astrocytes, and oligodendrocytes [1], [2]. NSCs have been found to persist in the adult brain and generate new neurons throughout adult life, particularly in the subgranular zone (SGZ) of the dentate gyrus and the subventricular zone (SVZ) of the lateral ventricles [3]. This raises the exciting possibility that NSCs may be useful for the therapy of neurodegenerative diseases. The factors that control the division and differentiation of NSCs are of tremendous scientific and medical importance.

Geminin (*Gmnn*) is an unstable regulatory protein that is thought to maintain neural progenitor cells in an undifferentiated state while they proliferate [4]. Geminin is expressed in both embryonic and adult mouse neural progenitor cells, and in the Xenopus central nervous system throughout embryonic development [5], [6]. Geminin is preferentially expressed in neural precursor cells, and expression is down-regulated before neural differentiation [7] [6]. Geminin binds to Brg1, the catalytic (ATPase) subunit of a SWI/SNF chromatin remodeling complex, and inhibits its recruitment to neuron-specific promoters by the basic helix-loop-helix (bHLH) transcription factors Neurogenin (Ngn) and NeuroD [7]. A complex between Geminin and the transcription factor AP4 represses the transcription of neuronal genes in non-neuronal cell types [8]. In addition to its effects on the nervous system, Geminin inhibits tissue differentiation in a variety of other organs by binding and inhibiting various transcription factors and chromatin remodeling proteins, including *sine oculis (Six)* and *Homeobox* (*Hox*) transcription factors and the *Polycomb* protein Scmh1 [7], [9], [10].

In addition to regulating cell differentiation, Geminin also limits the extent of DNA replication to one round per S phase by binding and inhibiting the essential replication factor Cdt1 [11]. The concentration of Geminin is cell-cycle regulated; the protein begins to accumulate at the G1/S transition and persists throughout S and G2 phase. Geminin is destroyed by ubiquitin-dependent proteolysis during M phase, which allows a new round of replication in the next cell cycle [12]. This expression pattern has been documented extensively in developing mouse brains [6]. *Six* and *Hox* transcription factors can compete with Cdt1 for binding to Geminin [9], [10], raising the possibility that Geminin links exit from the cell cycle with cell differentiation. According to this model, the destruction of Geminin when cells enter G0 phase would relieve the repression of Brg1 and other transcription proteins and trigger terminal differentiation [4], [13], [14].

In early embryos Geminin can also act as an inducer of nervous tissue. In an unbiased expression-cloning screen, Geminin was identified as a molecule that expands the size of neural plate in Xenopus embryos [5]. These effects are correlated with increased expression of the proneural gene Neurogenin-related 1 (Ngr1) and decreased expression of BMP4, an epidermis-inducing growth factor. Over-expression of Geminin in Drosophila embryos induces ectopic neural cells in the epidermis [15].

The role of Geminin in regulating neural development has been examined by deleting its gene from model organisms. C. elegans embryos treated with Geminin siRNA show gonadal abnormalities and ∼20% of the worms are infertile, but no neural phenotype has been described [16]. *Geminin^Δ/Δ^* Drosophila embryos die at larval stages with mostly normal neuroanatomy, although a small percentage of them have sharply reduced numbers of peripheral neurons [15]. Geminin-deficient Xenopus and mouse embryos do not develop past the blastula stage because of defects in DNA replication. Geminin-depleted Xenopus embryos arrest cell division in G2 phase at the mid-blastula stage because over-replication activates the DNA replication checkpoint [17], [18]. *Gmnn^Δ/Δ^* mouse embryos arrest development at about the 8-cell stage, as soon as the maternal supply of Geminin is exhausted [19], [20]. Their cells contain more nuclear DNA than normal, consistent with over-replication of the DNA. Interestingly, the *Gmnn^Δ/Δ^* cells prematurely differentiate as trophoblast cells and none express markers of the embryonic stem cells that form the embryo proper. *Gmnn(−)* Xenopus and mouse embryos arrest development long before neural induction takes place, which has precluded examining the role of Geminin in vertebrate neural development using a rigorous genetic system.

To address this question, we constructed a strain of mice in which Geminin was specifically deleted from neural stem cells. To our surprise, we found that neural-specific *Geminin^Δ/Δ^* mice displayed no obvious neurological defects and had apparently normal neurogenesis. We conclude that Geminin is dispensable for normal neurogenesis during most of embryogenesis and in adulthood.

## Results

The mouse genome contains a single copy of the Geminin gene, which is composed of seven exons. Exons 5, 6, and 7 encode Geminin's dimerization domain and the domains that bind Cdt1 and Brg1 (Figure S1). Because these domains are essential for Geminin's biological activity [21], deletion of these exons is predicted to produce a *Geminin^null^* allele. We flanked exons 5, 6, and 7 with loxP sites to create a floxed Geminin allele (*Gmnn^fl^*) and established a line of *Gmnn^fl/fl^* mice, which are completely viable and fertile [22]. To delete Geminin specifically from nerve cells, *Gmnn^fl/fl^* mice were crossed to *Nestin-Cre* mice. Nestin is a neurofilament protein that is expressed in neural precursor cells and NSCs [23]. *Nestin-Cre* mediated recombination begins around embryonic day 7.5 (e7.5), the time when the neural plate first forms, and continues throughout adulthood. Recombination is virtually complete in all neurons and glial cells by e15 [24], [25].

**Figure 1:**
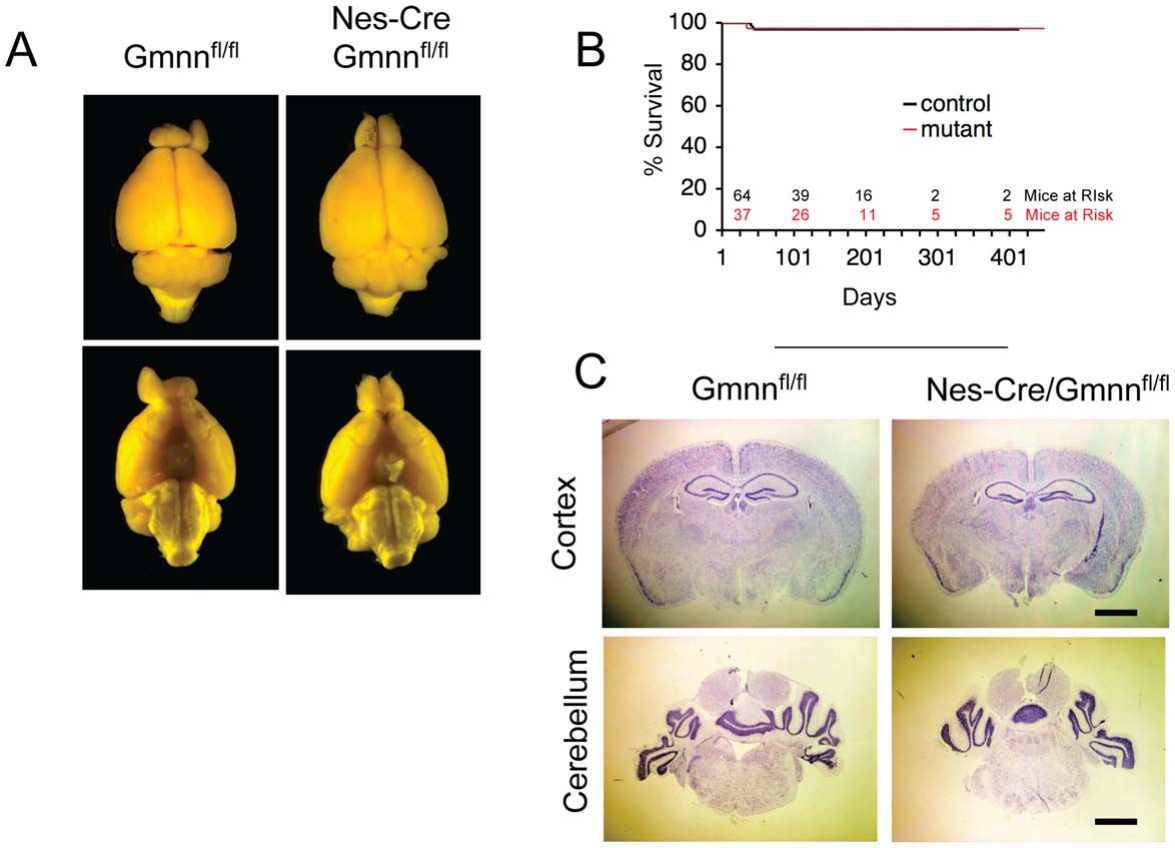
Nes-Cre/Gmnn^fl/fl^ Mice have Normal Neuroanatomy and Normal Survival. (A) Whole-mount brains of *Gmnn^fl/fl^* (left) and *Nes-Cre/Gmnn^fl/fl^* (right) mice. (B) Survival of *Gmnn^fl/fl^* (black) and *Nes-Cre/Gmnn^fl/fl^* (red) mice. Numbers above the X axis indicate the number of mice that were at risk at each timepoint (black, control animals; red, *Nes-Cre/Gmnn^fl/fl^* animals). Controls include both *Gmnn^fl/fl^* and *Gmnn^fl/+^* animals. (C) Representative Nissl-stained sections of the cortex and the cerebellum of *Gmnn^fl/fl^* and *Nes-Cre/Gmnn^fl/fl^* mice. Scale bar, 2 mm.

*Nestin-Cre/Gmnn^fl/fl^* mice were born in the expected Mendelian ratio (Table 1) and were indistinguishable from their control littermates in terms of size, activity, and longevity (Figure 1B). They moved all four extremities and exhibited normal locomotor activity. Their feeding and avoidance behavior were both normal, and they responded normally to noxious stimuli. Both males and females were able to mate and rear pups until the time of weaning, although the average litter size was slightly reduced when either parent had the genotype *Nestin-Cre/Gmnn^fl/fl^* or if the male had the genotype *Nestin-Cre/Gmnn^fl/+^* (Table 2). The brains of *Nestin-Cre/Gmnn^fl/fl^* mice were grossly normal in appearance and had no obvious neuroanatomical defects upon sectioning (Figure 1A and 1C).

**Table 1:** Viability of Nestin-Cre/Gem^fl/fl^ Mice.

	Offspring				
Parents	*Nestin-Cre Gem(+/loxP)*	*Gem(+/loxP)*	*Gem(loxP/loxP)*	*Nestin-Cre Gem(loxP/loxP)*	P Value (χ^2^)
*Nes-Cre/Gem^+/fl^ X Gem^fl/fl^*	22	21	27	28	0.82
*Nes-Cre/Gem^fl/fl^ X Gem^fl/fl^*	--	--	42	33	0.75

**Table 2:** Fertility of Nestin-Cre/Gmnn^fl/fl^ Mice.

Genotype	Sex	Litter size (mean ± SD)	# of Litters	# of Animals	P value
Control	M or F	7.8±2.0	19	6	--
*Nes-Cre/Gnmm(fl/+)*	F	7.4±1.3	5	3	0.700
*Nes-Cre/Gnmm(fl/+)*	M	5.3±2.0	9	4	0.007
*Nes-Cre/Gnmm(fl/fl)*	F	5.2±2.9	15	4	0.002
*Nes-Cre/Gnmm(fl/fl)*	M	5.3±2.6	19	3	0.006

**Figure 2:**
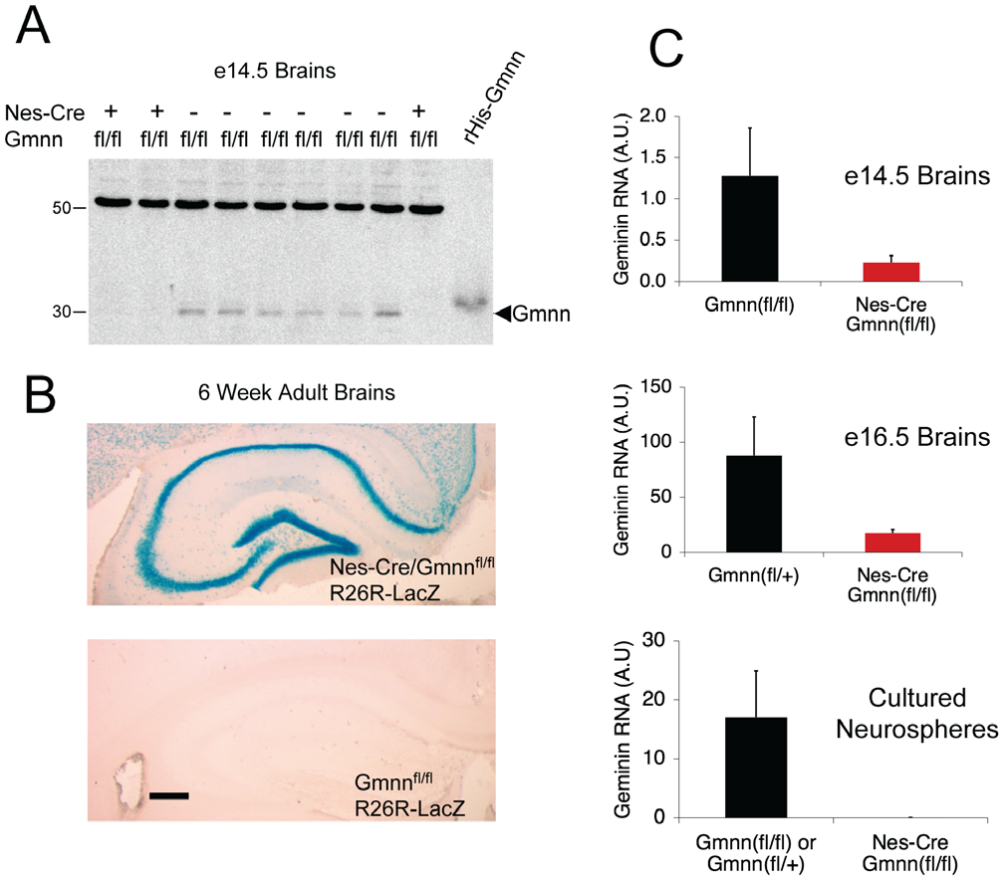
Geminin is Deleted from the Brains of Nes-Cre/Gmnn^fl/fl^ Mice. (A) Immunoblot showing the amount of Geminin protein in e14.5 mouse brain homogenates. (B) Xgal staining of coronal brain sections showing β-galactosidase activity in the brains of *Nes-Cre/Gmnn^fl/fl^/R26R-LacZ* and *Gmnn^fl/fl^/R26R-LacZ* mice. Scale bar, 0.25 mm. (C) Quantitative real-time PCR showing the amount of Geminin mRNA in the brains of e14.5 and e16.5 mice and in neurospheres cultured from newborn (P0) mice.

To confirm that the Geminin gene had been deleted, we isolated protein and RNA samples from the brains of e14.5 animals. Geminin protein was undetectable in the brains of *Nestin-Cre/Gmnn^fl/fl^* mice compared to the brains of littermate controls (Figure 2A). The brains of e14.5 and e16.5 *Nestin-Cre/Gmnn^fl/fl^* mice had only ∼20% of the level of intact Geminin mRNA found in littermate controls as judged by quantitative RT-PCR (Figure 2C). We hypothesized that the residual amount of Geminin message may have come from Geminin expression in vascular or other non-neural cell types. To see if this was the case, we cultured neurospheres from the brains of newborn mice and measured Geminin mRNA levels in these purified NSC populations. The level of intact Geminin RNA in *Nestin-Cre/Gem^fl/fl^* neurospheres was only ∼0.2% of the concentration in control neurospheres, which indicates virtually complete deletion of the Geminin gene (Figure 2C, bottom panel). As an additional control we crossed our mice to *R26R-LacZ* mice, in which a loxP-flanked transcription/translation STOP cassette is inserted between the ROSA promoter and the β-galactosidase gene [26]. *Cre*-mediated recombination removes the STOP cassette and causes β-galactosidase expression in cells that express *Cre* and their descendants. The brains of adult (6–8 week old) *Nestin-Cre/Gem^fl/fl^/R26R-LacZ* mice showed β-galactosidase expression in virtually all neurons while the brains of littermate *Gmnn^fl/fl^/R26R-LacZ* mice showed no β-galactosidase expression (Figure 2B). Taken together, our results indicate that deletion of the Geminin gene is mostly complete by e14.5 and virtually complete by birth, consistent with what has been previously reported for other mice that express *Nestin-Cre* [24], [25]. The persistence of β-galactosidase-expressing neurons in the brains of adult *Nestin-Cre/Gmnn^fl/fl^/R26R-LacZ* mice excludes the possibility that neurogenesis is rescued by rare cells that escape Cre-mediated Geminin deletion.

**Figure 3:**
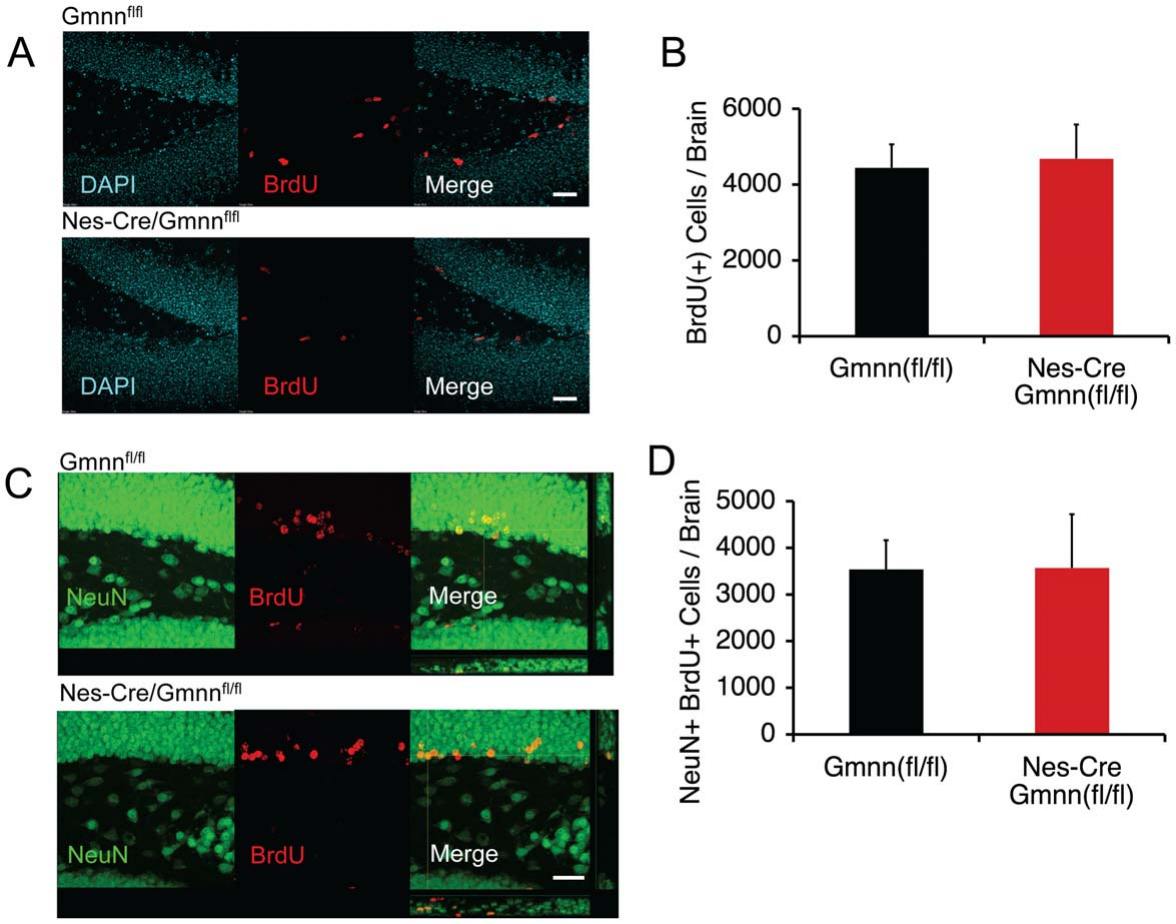
Normal Hippocampal Neurogenesis in Nes-Cre/Gmnn^fl/fl^ Mice. (A) Adult mice were injected with BrdU 24 hours before sacrifice. Adjacent sections of the hippocampus were stained with either anti-BrdU antibodies or with DAPI. Scale bar, 50 µM. (B) Quantification of the number of BrdU(+) cells in the subgranular zone of the hippocampus in *Nes-Cre/Gmnn^fl/fl^* mice and control littermates. n = 4 for controls; n = 3 for *Nes-Cre/Gmnn^fl/fl^* mice. (C) 7 week-old mice were injected with BrdU for 12 days then sacrificed 9 weeks later. Sections of the hippocampus were stained with anti-BrdU antibodies and with anti-NeuN antibodies. The Z-stack image on the right shows BrdU^+^, NeuN^+^, and BrdU^+^NeuN^+^ cells. Scale bar, 50 µM. (D)Quantification of the number of BrdU(+)NeuN(+) cells in the hippocampus of *Nes-Cre/Gmnn^fl/fl^* mice and control littermates. n = 2 for both genotypes.

Next we examined the process of neurogenesis in more detail to see if we could detect quantitative defects in neuron formation. First we compared the number of NSCs present in the SGZ of the dentate gyrus of 2-month old *Nestin-Cre/Gmnn^fl/fl^* and control mice. The mice were injected with bromodeoxyuridine (BrdU) and 24 hours later the brains were fixed, sectioned, and stained with anti-BrdU antibodies. There was no significant difference in the number of BrdU^+^ NSCs in the dentate gyri of *Nestin-Cre/Gmnn^fl/fl^* and control mice (Figure 3A and 3B). We also performed a pulse-chase experiment to monitor the differentiation of NSCs into neurons. Adult 7 week-old mice were injected daily with BrdU for 12 days, then 9 weeks later their brains were fixed, sectioned, and stained with antibodies against BrdU and the neuronal marker NeuN (Figure 3C). We counted the number of newly born neurons (NeuN^+^BrdU^+^ cells) in the dentate gyrus using confocal microscopy. We found no difference in the number of BrdU^+^NeuN^+^ cells in the dentate gyri of *Nestin-Cre/Gmnn^fl/fl^* or control mice. These results indicate that in *Nestin-Cre/Gem^fl/fl^* brains the number of NSCs is normal and their rate of differentiation into neurons is normal.

**Figure 4:**
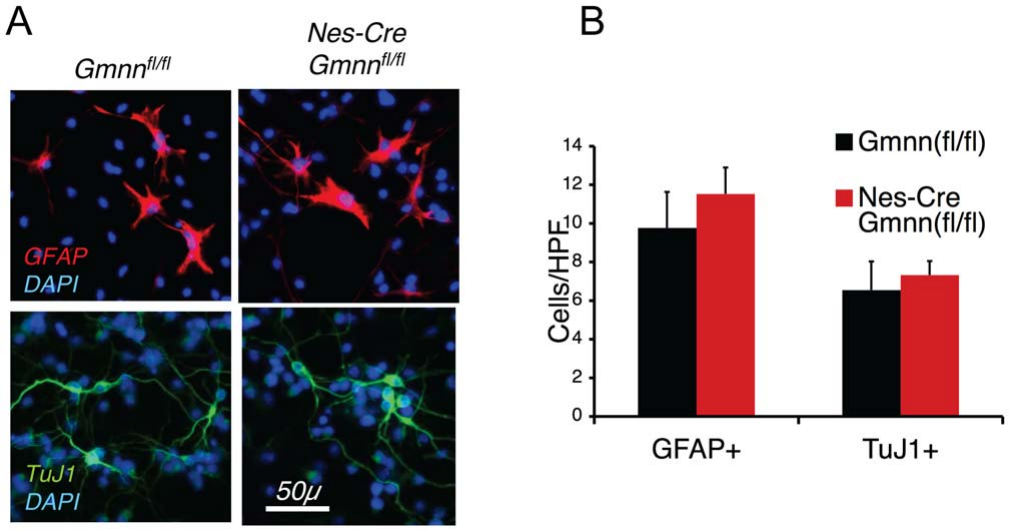
Normal Differentiation of *Nes-Cre/Gmnn^fl/fl^* Neurosphere Cells. (A)Representative images of TuJ1(+) neurons and GFAP(+) astrocytes derived from *Nes-Cre/Gmnn^fl/fl^* and control neurosphere cells. Scale bar, 50 µM. (B)Quantification of the number of TuJ1(+) neurons and GFAP(+) astrocytes derived from *Nes-Cre/Gmnn^fl/fl^* and control neurosphere cells. HPF, High Powered Field.

To test whether Geminin regulates the differentiation of NSCs in vitro, we used primary neurosphere cultures from neonatal *Nestin-Cre/Gmnn^fl/fl^* and control brains. Neurosphere cultures could easily be established from both genotypes. To induce differentiation, neurospheres were cultured in a low concentration of growth factors then stained with specific antibodies to identify differentiated neurons (TuJ1^+^ cells) or astrocytes (GFAP^+^ cells). We found that *Nestin-Cre/Gmnn^fl/fl^* and control neurospheres produced equal numbers of GFAP^+^ and TuJ1^+^ cells (Figure 4).

**Figure 5:**
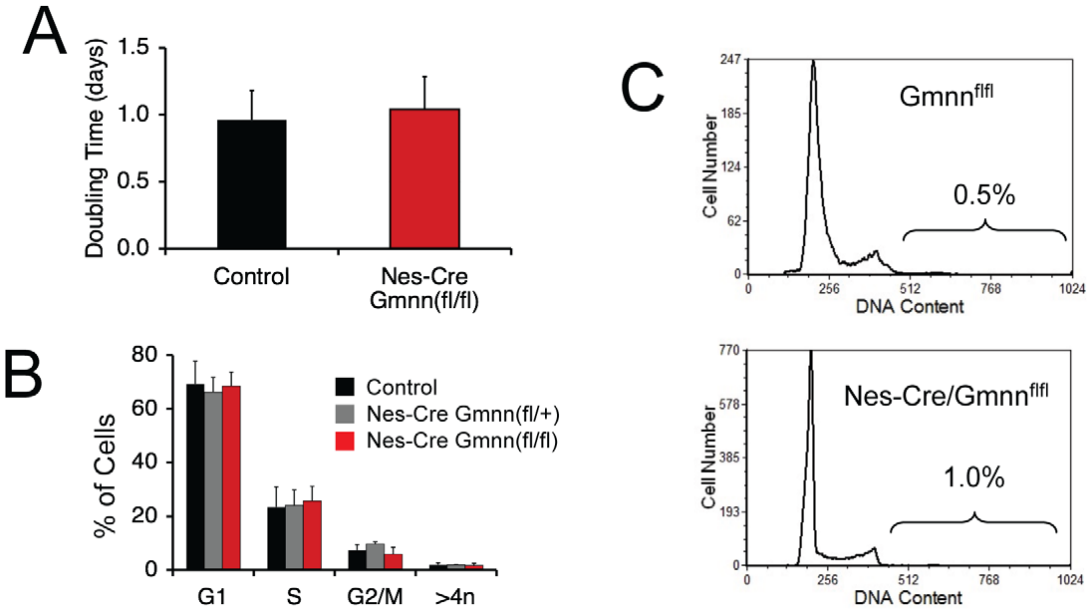
*Nes-Cre/Gmnn^fl/fl^* Neurosphere Cells Show Normal DNA Replication and Normal Cell Cycle Kinetics. (A)Doubling time, (B) cell cycle phase distribution, and (C) DNA content of neurosphere cells derived from *Nes-Cre/Gmnn^fl/fl^* mice and control littermates.

Finally, we examined the effect of Geminin deletion on the cell cycle in neurospheres. Both *Nestin-Cre/Gmnn^fl/fl^* and control neurospheres cells grew at identical rates (Figure 5A), and the distribution of cells in different phases of the cell cycle was indistinguishable between the two types of cells (Figure 5B). In particular, the percentage of *Nestin-Cre/Gmnn^fl/fl^* cells that had excessive DNA contents >4n was the same as in *Gmnn^fl/fl^* littermate controls, and both genotypes had the same proportion of cells with a G2/M DNA content (Figure 5C). These results indicate that *Gmnn^Δ/Δ^* neurosphere cells do not over-replicate their DNA and display no obvious cell cycle defects.

## Discussion

Geminin is widely expressed in the developing brain and is thought to have two functions in controlling the development of the central nervous system. Early in development, Geminin acts as a neural inducer: over-expression of the protein causes expansion of the neural plate at the expense of the epidermis [5]. Later in development, Geminin inhibits neuronal differentiation by inhibiting the recruitment of a SWI/SNF chromatin-remodeling complex to neuron-specific promoters [7]. Geminin also limits the extent of DNA replication during the cell cycle by binding and inhibiting the essential replication factor Cdt1 [27]. Because of its effects on both the cell cycle and on gene expression, it has been proposed that Geminin maintains neural stem cells in an undifferentiated state while they proliferate [4], [13], [14].

In this study we rigorously examined the role of Geminin in vertebrate neurogenesis by constructing a strain of *Nestin-Cre/Gmnn^fl/fl^* mice in which Geminin was deleted from neural stem cells early in embryonic development. To our surprise, we could detect no defects in neurogenesis in *Geminin^Δ/Δ^* brains. *Nestin-Cre/Gmnn^fl/fl^* mice had normal viability and displayed no obvious neurological or neuroanatomical abnormalities. Quantitative assays of neural stem cell division and differentiation patterns also revealed no abnormalities. Previous studies have shown that mice with defects in adult neurogenesis have well defined and unmistakable phenotypes. For example, mice that carry a NSC-specific deletion of the chromatin remodeling factor *Mll1* develop growth retardation and ataxia during the second week of life and die around 4 weeks of age [28]. Mice with a postnatal deletion of both *Numb* and *Numb-like* show defects in lateral ventricle integrity and SVZ neuroblast survival [29]. We conclude that Geminin is largely dispensable for adult neurogenesis and for embryonic neurogenesis after e14.5. Our results are consistent with what has been reported for *Geminin^Δ/Δ^* Drosophila larvae, most of which have normal neural architecture [15].

It is unlikely that the mouse genome encodes a protein that can substitute for Geminin, which is a single copy gene. The 41 kD GEMC1 protein (GEMinin Coiled-coil domain containing protein 1) includes a 49 amino-acid sequence that is ∼30% identical to Geminin's coiled coil, but GEMC1 serves a different function than Geminin and is actually required for DNA replication [30]. Mice carrying a homozygous deletion of the Geminin gene die at the early blastula stage, as soon as the maternal supply of Geminin is exhausted [19], [20]. We have also observed strong phenotypes when Geminin is deleted from other types of cells using other types of *Cre* drivers. For example, deleting Geminin profoundly affects the differentiation pattern of bone marrow cells; the production of red blood cells is virtually abolished while the production of megakaryocytes is greatly expanded [22].

Geminin's role in controlling the extent of DNA replication has been well documented by many different laboratories using many different experimental systems [11], [27]. It is therefore somewhat surprising that DNA replication and cell division appear completely normal in *Gmnn^Δ/Δ^* neurosphere cells. These cells probably cycle normally in the absence of Geminin because of redundant Geminin-independent mechanisms that limit the extent of DNA replication. For example, in addition to being inhibited by Geminin, Cdt1 is also destroyed by ubiquitin-dependent proteolysis during S phase, and the degradation is coupled to the initiation of DNA replication[31]. In order to induce over-replication in Xenopus egg extracts, it is necessary to both remove Geminin and inhibit the proteolysis of Cdt1 [17], [31], [32]. The requirement for Geminin to prevent re-replication seems to vary depending upon the type of cell and upon conditions. For example, *Gmnn^Δ/Δ^* white blood cells over-replicate their DNA when stimulated with growth factors [22], and *Gmnn^Δ/Δ^* T lymphocytes show cell cycle defects when stimulated to proliferate in vitro [33].

Some of the previous studies that implicated Geminin in neurogenesis were based on over-expressing the protein, which may cause non-physiological effects [5], [15]. Others employed cultured cell lines, which may not reproduce all the characteristics of in vivo NSCs [7]. Some of the previously observed effects on neurogenesis may have been caused by cell cycle abnormalities rather than a specific effect of Geminin on neural differentiation.

It remains possible that Geminin is required for very early neural induction. The neural plate becomes visible as distinct anatomical structure at e7.5 and the neural tube becomes completely closed by e10 [34]. Both these events occur several days before *Nestin-Cre* mediated recombination is complete. Even so, *Nestin-Cre*-mediated recombination has been detected as early as e7.5, and extensive recombination has occurred in the midbrain and hindbrain by e9.5 [24], [25]. Our targeting construct did not delete the sequences encoding the small “neuralizing domain” found near Geminin's amino terminus. Expression of this fragment in Xenopus embryos induces neural tissue as efficiently as full-length Geminin [5]. Although we could detect transcripts encoding this domain in *Nestin-Cre/Gmnn^Δ/Δ^* neurospheres, we could not detect a new fusion protein in *Gmnn^Δ/Δ^ brains* (Figs. S2 and S3). Geminin deficiency might cause defective neurogenesis in strains with a different genetic background. It is also possible that *Nestin-Cre/Gem^fl/fl^* mice will prove to have a subtle neurological or neuroanatomical defect than could not be detected by the methods employed here. Such a defect might explain the mild decrease in fertility we observed in *Nestin-Cre/Gmnn^fl/fl^* mice. Nevertheless, our results clearly indicate that Geminin is largely dispensable for mammalian neurogenesis.

## Materials and Methods

### Ethics Statement

All animal work was performed according to the protocol approved by the Nothwestern University Animal Care and Use Committee (Protocol # 2009-0911).

### Mouse Breeding

The construction of the *Gmnn^fl^* allele was described previously [22]. *Nestin-Cre* mice (B6.Cg-Tg(Nes-Cre)1Kln/J, Jackson Labs Strain 3771) were kindly provided by Dr. Anjen Chenn (Northwestern University). For most experiments, *Nes-Cre/Gmnn^fl/fl^* mice were mated to *Gmnn^fl/fl^* mice to produce experimental *Nes-Cre/Gmnn^fl/fl^* mice and control *Gmnn^fl/fl^* littermates. To obtain embryos at defined embryonic stages, timed matings were performed in which male and female mice were caged together overnight then separated immediately after the mating plug appeared. Genotypes were determined by PCR of tail DNA. The following primers were used for amplification: Nes-Cre forward (5′-gcctgcattaccggtcgatgcaacga-3′), Nes-Cre reverse (5′-gtggcagatggcgcggcaacaccatt-3′), Geminin forward (5′-gctcagaggtttcaggg-3′), Geminin^WT^ reverse (5′-catcaggtgttctctcaagtgtctg-3′), and Geminin^fl^ reverse (5′-gctacttccatttgtcacgtcc-3′).

### Antibodies

Affinity-purified anti-mouse Geminin antibody was obtained by immunizing rabbits with recombinant his-Geminin and purifying antibodies from serum over a column of recombinant his-Geminin coupled to cyanogen bromide sepharose beads [22], [35].

### Real Time PCR

RNA was isolated from fresh tissue using Trizol reagent (Invitrogen). cDNA synthesis was carried using a standard kit (Ambion) and RT-PCR was performed using an Applied Biosystems 7500 Fast Real Time PCR System. Primers and fluorescently labeled probes for RT-PCR were designed using Primer Design software (Applied Biosystems). All RNA levels were normalized to the amount of 18S ribosomal RNA in each sample. For Figure 2, Geminin RNA primer sequences were 5′-acggatgctaggccgtgtac-3′ (forward), 5′-gcaccgtgtagttagtttaccaagag-3′ (reverse), and 5′-acgcactgccagcgttgccc-3′ (probe). 18S RNA primer sequences were 5′-aacgagactctggcatgctaact-3′ (forward), 5′-cgccacttgtccctctaagaa-3′ (reverse), and 5′-ttacgcgacccccgagcgg-3′ (probe). For PCR of exons 3 and 4 (Figure S3), Geminin primers were 5′-gtgaagaatagtcctgtccc-3′ (forward) and 5′-ccacagcttgaagtctgag-3′ (reverse). SYBR green was used instead of a probe.

### Histology and Immunohistochemistry

For pulse labeling, mice were injected intra-peritoneally with three doses of bromodeoxyuridine (Invitrogen B23151, 10 mg/ml in PBS, 50 mg/kg) separated by two hours. Brains were fixed 24 hours after the last BrdU dose by transcardial perfusion with a solution of 4% paraformaldehyde in PBS then post-fixed overnight at 4°C in the same solution. For pulse-chase analysis, mice received a single daily dose of BrdU (50 mg/kg) for 12 days and the brains were fixed 9 weeks after the final dose. Free-floating coronal sections (40 µM) were cut using a vibratome (Leica VT 1000S) and collected in cold PBS. For β-galactosidase staining, sections were incubated at room temperature in PBS containing 5 mM K_3_Fe(CN)_6_, 5 mM K_4_Fe(CN)_6_, 2 mM MgCl_2_, and 1 mg/ml X-gal (5-bromo-4-chloro-3-indolyl-β-D-galactopyranoside, Roche). For Nissl staining, paraformaldehyde-fixed brains were embedded in OCT Medium and cut into frozen sections (10 µM). Nissl staining was performed using standard procedures [36]. To stain for BrdU, sections were incubated in 0.1 N HCl in PBS at 60°C for 20 minutes then washed three times in PBS at room temperature. Sections were blocked in 1X PBS, 10% Normal Donkey Serum (NDS, Jackson Immuno Research), 0.5% Triton X-100 (TX-100) and stained overnight at 4°C with anti-BrdU antibody (Fitzgerald Industries #20-BS17) diluted 1:500 in 1X PBS, 7.5% NDS, and 0.25% TX-100. After extensive washing in PBS, sections were sequentially stained with biotinylated Donkey anti-Sheep antibody (Jackson #713-065-003) diluted in 1X PBS, 5% NDS, and 0.25% TX-100 and with Alexa 663-conjugated streptavidin (Invitrogen S-21375) diluted in 1X PBS. For NeuN staining, BrdU-stained sections were blocked in 1X PBS, 4% Normal Goat Serum (NGS, Jackson), and 0.25% TX-100 then stained overnight at 4°C with anti-NeuN antibody (Millipore clone A60, #MAB377) diluted 1:300 in 1X PBS, 2% NGS, and 0.25% TX-100. After washing in PBS, the sections were stained with Alexa 488-conjugated Donkey anti-Mouse IgG1 (Invitrogen A21121) diluted 1:300 in 1X PBS, 1% NDS, and 0.25% TX-100. Sections were mounted in Vectashield medium containing DAPI (Vector Laboratories). To determine the number of BrdU^+^ cells in the dentate gyrus, we counted every sixth section (8 sections per brain) and multiplied the result by 6. A total of 900 -1400 BrdU^+^ cells were counted per brain. To determine the number of NeuN^+^BrdU^+^ double-positive cells, we took confocal images of representative sections and used Z-stack images to determine the percentage of BrdU^+^ cells that were also NeuN^+^, then multiplied this percentage (∼50% for all mice) by the total number of BrdU^+^ cells. To determine these percentages we counting an average of 250 BrdU^+^ cells per brain.

### Neurosphere Culture and Differentiation

Primary neurosphere cultures were obtained from newborn (P0) mice as described [37], [38], [39]. Neurospheres were grown in DMEM/F-12 medium containing N2 supplement, B27 supplement, penicillin, streptomycin, glutamine (all from Invitrogen), and 20 ng/ml Epidermal Growth Factor (EGF, BD Biosciences). After 3 days the cells were dissociated with trypsin, triturated, counted, and re-seeded at a density of 5 X 10^4^ cells/ml. To calculate the doubling time, cells were trypsinized and re-counted three days later. For cell cycle analysis the cells were stained with propidium iodide using standard procedures[40]. To induce differentiation, dissociated neurosphere cells were seeded onto poly-D-lysine coated coverslips (Sigma, 20 µg/ml for >1 hour) in medium containing 1 ng/ml EGF. The medium was changed after 3 days, and after 7 days the coverslips were washed with PBS and fixed in 1X PBS/4% paraformaldehyde. For antibody staining, coverslips were washed with 1X PBS/0.1% TX-100 (PBST) then incubated overnight at 4°C in PBS/5% NGS containing either anti-TuJ1 antibody (Mouse IgG2b, clone SDL.3D10, Sigma #T8660) or anti-GFAP antibody (Mouse IgG1, clone GA5, Sigma # G3893). The secondary antibodies were Alexa 488-conjugated goat anti-mouse IgG2b (Invitrogen # A21141) and Cy3-conjugated goat anti-rabbit IgG (Jackson Immuno Research #111-165-144) respectively.

## Supporting Information

Figure S1 **Protein Domains Deleted in** ***Gmnn^Δ/Δ^*** **Mice.** (Top) Map of the Geminin locus with color-coded exons. Deleted exons are enclosed by the rectangle. (Bottom) Exon boundaries mapped onto the domains of the Geminin protein. D-box, destruction box; NLS, bipartite Nuclear Localization Signal; Neural, neuralizing domain (underlined) which overlaps the D-box and the NLS.
(TIF)

Figure S2 **Geminin Deletion does not Generate a Detectable New Fusion Protein.** The full immunoblot from Figure 2A. Biotinylated MW markers are shown on the left.
(TIF)

Figure S3 **Geminin Deletion does not Cause Over-Expression of Geminin RNA.** RNA was isolated from undifferentiated or differentiated neurospheres from control or Nes-Cre/Gmnn(fl/fl) mice. The amount of exon 3/4-containing RNA was determined by RT-PCR. The location of the amplified fragment is indicated in Figure S1.
(TIF)
