# Supplementary figures and images for: Geminin-Deficient Neural Stem Cells Exhibit Normal Cell Division and Normal Neurogenesis
Source: PLoS One. 2011 Mar 9;6(3):e17736. doi: 10.1371/journal.pone.0017736 (PMC3052383; doi:10.1371/journal.pone.0017736)

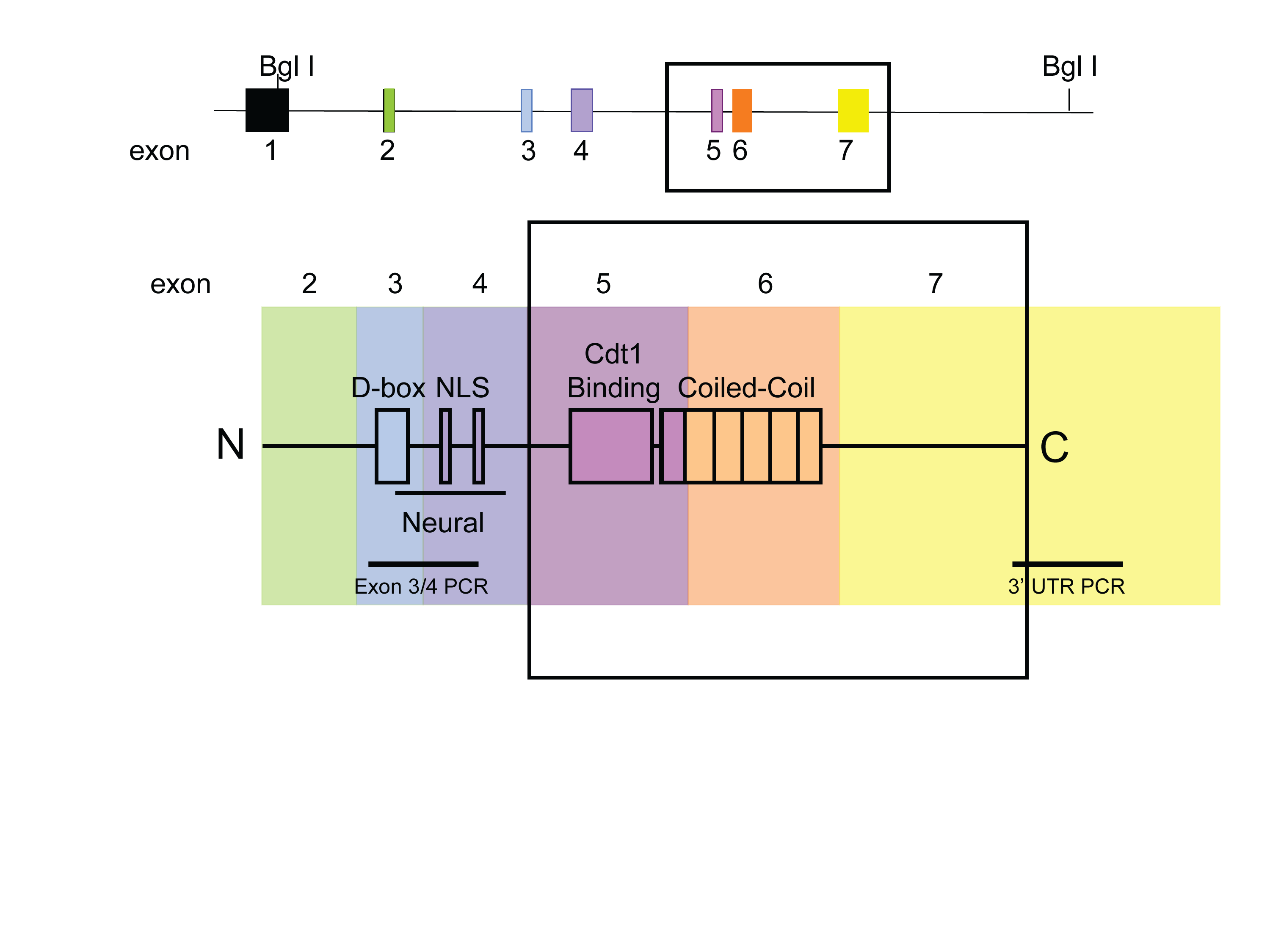

Supplement: Figure S1 — Protein Domains Deleted in GmnnΔ/Δ Mice. (Top) Map of the Geminin locus with color-coded exons. Deleted exons are enclosed by the rectangle. (Bottom) Exon boundaries mapped onto the domains of the Geminin protein. D-box, destruction box; NLS, bipartite Nuclear Localization Signal; Neural, neuralizing domain (underlined) which overlaps the D-box and the NLS. (TIF) [file pone.0017736.s001.tif]

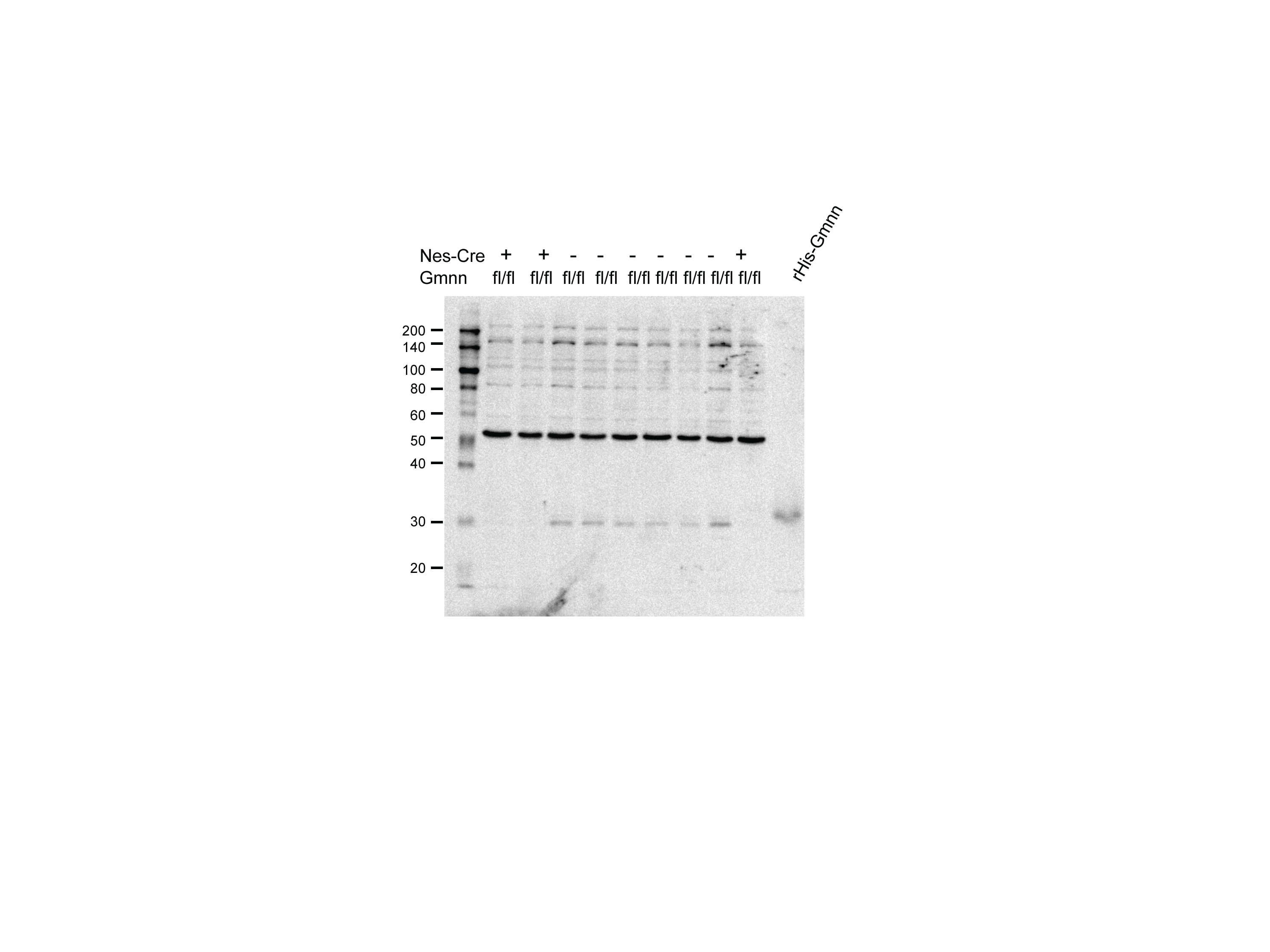

Supplement: Figure S2 — Geminin Deletion does not Generate a Detectable New Fusion Protein. The full immunoblot from Figure 2A. Biotinylated MW markers are shown on the left. (TIF) [file pone.0017736.s002.tif]

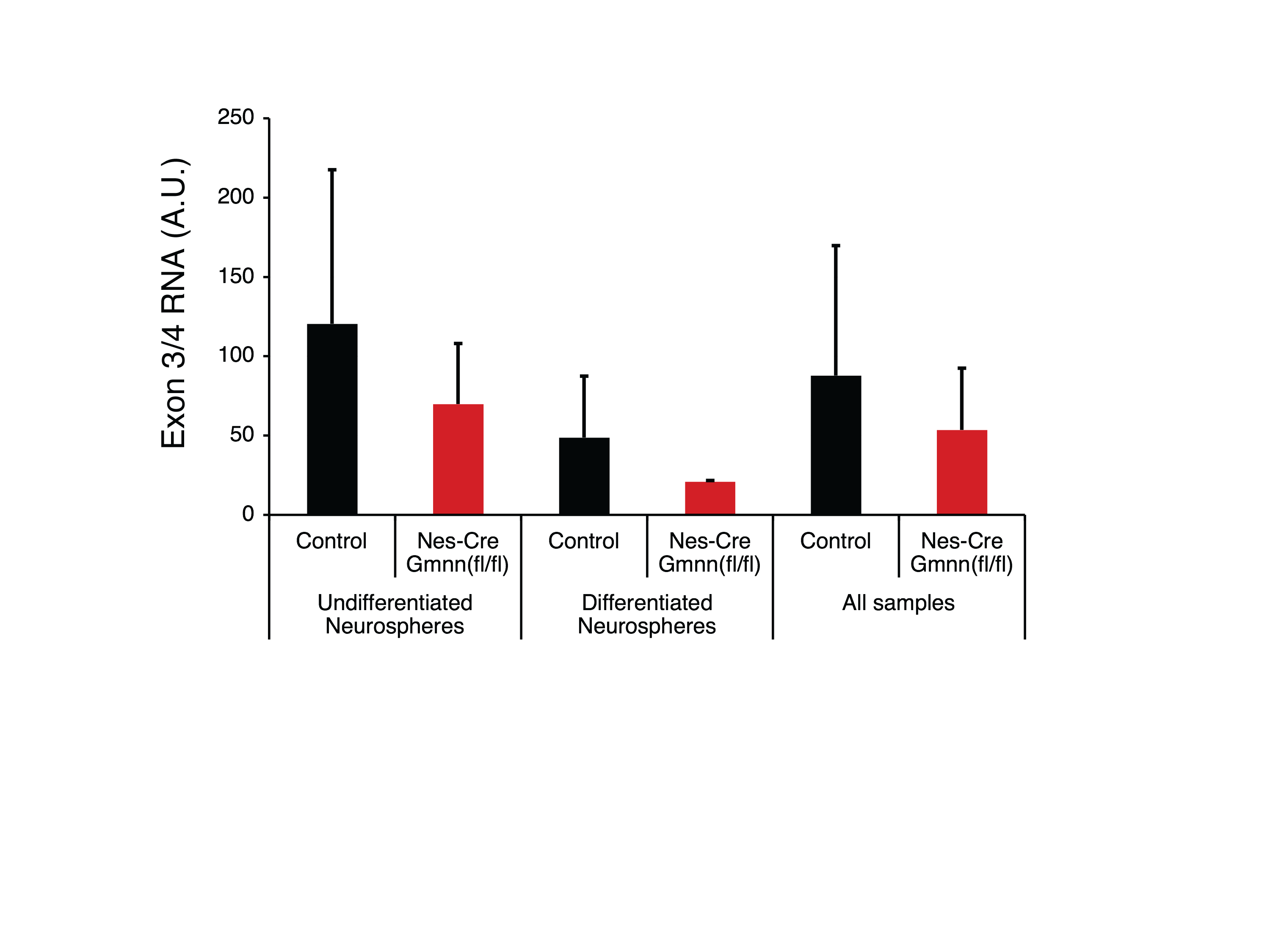

Supplement: Figure S3 — Geminin Deletion does not Cause Over-Expression of Geminin RNA. RNA was isolated from undifferentiated or differentiated neurospheres from control or Nes-Cre/Gmnn(fl/fl) mice. The amount of exon 3/4-containing RNA was determined by RT-PCR. The location of the amplified fragment is indicated in Figure S1. (TIF) [file pone.0017736.s003.tif]
